# Supplementary material for: Anapole-state-enhanced 2D chiral photodetector operating in the near-infrared second window
Source: Nat Commun. 2026 Feb 18;17:2907. doi: 10.1038/s41467-026-69727-z (PMC13031947; doi:10.1038/s41467-026-69727-z)
Supplement: Supplementary file 1 — Supplementary Information [file 41467_2026_69727_MOESM1_ESM.pdf]

Supplementary Information for "Anapole-state-enhanced 2D chiral photodetector operating in the near-infrared second window"

*Qi-hang Zhang[1,2,3,4,#], Zi-hao Dong[1,2,3,4,#], Kai Liu[1,5], Shao-jie Fu[1,5], Xu-hao Hong [1,5], Yu-lin Cao[6], Chao Zhang[1,2,3,4], Jun Du[1,5], Yan-qing Lu[1,2,3,4], Yong-yuan Zhu[1,5], Yan-feng Chen[1,2,3,4], and Xue-jin Zhang[1,2,3,4*]*

[1]State Key Laboratory of Solid State Microstructures, and Collaborative Innovation Center of Advanced Microstructures, Nanjing University, Nanjing, 210093, China
[2]Key Laboratory of Intelligent Optical Sensing and Manipulation, Nanjing University, Nanjing, 210093, China
[3]Jiangsu Key Laboratory of Artificial Functional Materials, Nanjing University, Nanjing, 210093, China
[4]College of Engineering and Applied Sciences, Nanjing University, Nanjing, 210093, China
[5]School of Physics, Nanjing University, Nanjing, 210093, China
[6]Physics Laboratory, Industrial Training Center, Shenzhen Polytechnic University, Shenzhen 518055, China
[#]These authors contributed equally to this work.

*e-mail: xuejinzh@nju.edu.cn

Supplementary Note 1. The fabrication process of the device

Supplementary Note 2. Period of the metasurface

Supplementary Note 3. Optimization of the parameters for the symmetrical structure

Supplementary Note 4. Modulation of the asymmetry of the structure

Supplementary Note 5. Identification of the BIC mode

Supplementary Note 6. Construction of anapole states

Supplementary Note 7. Electric measurements of $MoS_2$, $WSe_2$ monolayers and the heterostructure

Supplementary Note 8. PL spectra of the 2D TMDCs

Supplementary Note 9. Influence of the 2D material on the optical property of metasurface

Supplementary Note 10. Advantages of the single-crystalline Ag

Supplementary Note 11. Consistency of the enhancement of SHG and TPA

Supplementary Note 12. SHG spectra for heterostructure on metasurface

Supplementary Note 13. Power-dependent optical and photoelectric signals

Supplementary Note 14. Component analysis of the enhanced photocurrent

Supplementary Note 15. Noise measurement and detectivity assessment

Supplementary Note 16. Photoconductive gain mechanism

Supplementary Note 17. Regulation of Schottky barrier by gate voltage

Supplementary Note 18. Polar plots of SHG

Supplementary Note 19. Regulation of the vertically polarized mode

Supplementary Note 20. Chiral measurement of the structure with mirror symmetry

Supplementary Note 21. Influence of the angle of the cruciform structures

Supplementary Note 22. Modulation of the mirror asymmetry

Supplementary Note 23. Angle-resolved spectra of the chiral structure

Supplementary Note 24. Multipole decomposition of the chiral-resolved structure

Supplementary Note 25. Chiral measurements of SHG

Supplementary Note 26. Comparison of photoresponse performances

Supplementary Note 27. Photoelectric measurement platform

## Supplementary Note 1. The fabrication process of the device

Single-crystalline Ag was synthesized by a polyol reduction method and sprinkled onto the $SiO_2$/Si substrate. Lithography technique was applied to define the patterns, then followed by the $SiO_2$ and Au deposition using magnetron sputtering (Gatan 682). The thickness of single-crystalline Ag was 1-2 μm. To ensure the pad film continuity between top of single-crystalline Ag and $SiO_2$/Si substrate, the edges of the single-crystalline Ag were cut by FIB and covered by a thick $SiO_2$ film of 200 nm. The other $SiO_2$ film with thickness of 100 nm positioned between Ag substrate and Au electrodes acted as an insulation layer. Then, the nanostructure was etched on the surface of the Ag by FIB to construct metasurface. After transferring $WSe_2$ and $MoS_2$ sequentially on Ag metasurface with PDMS, top gate electrode formed by Au (30 nm)/$SiO_2$ (100 nm) was fabricated on top of $MoS_2$/$WSe_2$ heterostructure.

For dielectric substrates, Au electrodes were pre-patterned on $SiO_2$/Si, then $MoS_2$ and $WSe_2$ were transferred onto top of $SiO_2$/Si by PDMS.

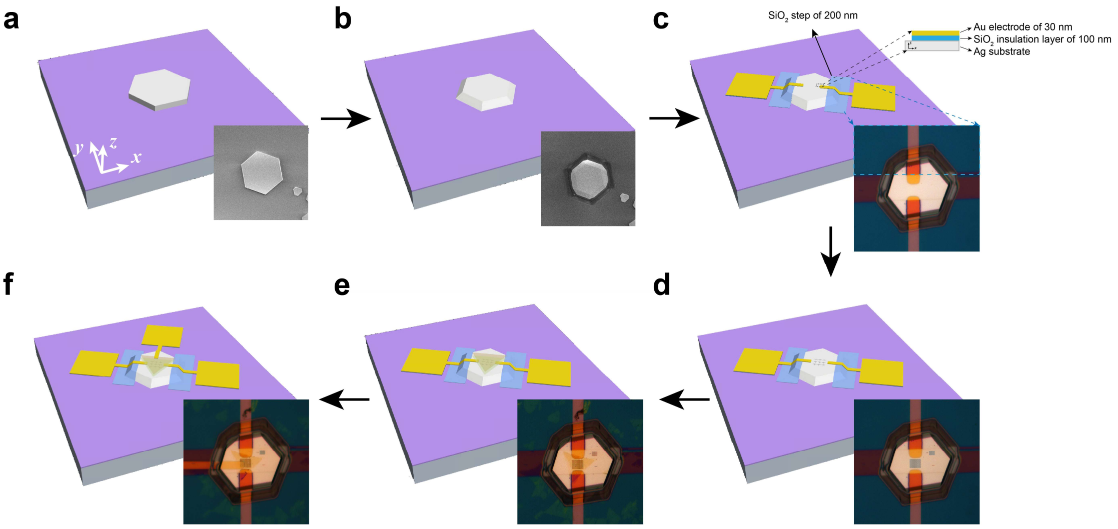


**Supplementary Fig. 1 | Fabrication process of $MoS_2$/$WSe_2$ heterostructure photodetector on Ag metasurface.**

For the stability of the single-crystalline Ag, it is coated by polyvinyl pyrrolidone (PVP) during and after synthesis, offering the Ag superior oxidation and sulfuration resistance, allowing it to remain stable in air for a duration sufficient for device fabrication. After fabrication, the structures are immediately encapsulated with transferred 2D materials to isolate them from air, enabling long-term stability for photoelectric characterization. Our fabricated devices have been successfully stored in low-humidity air for over one month without performance degradation.

**Supplementary Note 2. Period of the metasurface**

For the mode that the mode wavelength that shorter than that of the SPP mode, the mode resonance will be rather weak,[1] as shown in Fig. S2. In order to achieve a broadband optical response in NIR-II region in the structure, the period is set as 1000 nm.

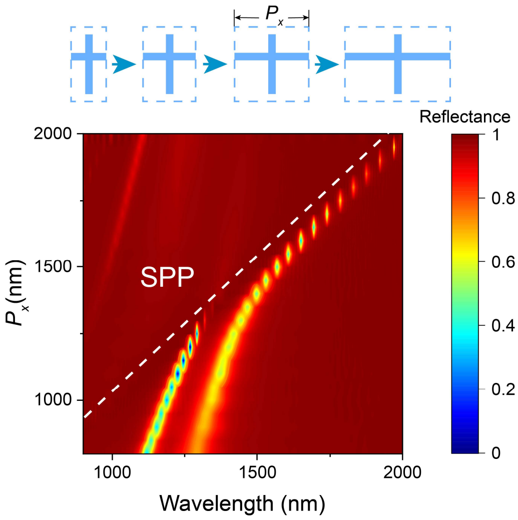


**Supplementary Fig. 2 | Simulated reflectivity spectra of the structure with the period of $P_x$.**

**Supplementary Note 3. Optimization of the paraments for the symmetrical structure**

Figure S3a shows the schematic of the unit cell of the metasurface at $\Delta y = 0$. For an excellent performance among the near-infrared region, the mode wavelength of SPP should shorter than this region,[1] as shown in Fig. S2. Then the period is set as $P = 1000$ nm. Under horizontally polarized illumination, the GPP mode is supported by the vertical groove of the structure. The mode wavelength of the GPP mode red shifts with the $L_V$ and $d$, and blue shifts with the $w$. In order to achieve a great performance at the wavelength of 1550 nm, these paraments should coordinate with each other. Restricted by the period, the $L_V$ is set as 800 nm.

Theoretical SHG and TPA EF, defined as EF = average $|\boldsymbol{E}_{\mathbf{en}}|^4/|\boldsymbol{E}_0|^4$, where $\boldsymbol{E}_0$ is the electric field of incident light, and $\boldsymbol{E}_{\mathbf{en}}$ is the enhanced electric field among the structure, is used to compare the enhancement performance of the metasurface. For a certain $w$, the mode wavelength red shifts with the $d$, and the enhancement performance is increased firstly and then goes down, as shown in Fig. S3b,c. Changing the $d$ for each $w$, as shown in Fig. S3d, the structures with different $w$s exhibit different trends by the $d$, and each $w$ corresponds to an optimal $d$. Putting eyesight on the wavelength, it can be found that there is an optimal $w$ for each wavelength, as shown in Fig. S3e. For the shorter wavelength, it may follow the law that narrower $w$ brings larger enhancement. However, for the interested region of near 1550 nm, the $w$ of 100 nm shows obvious advantages.

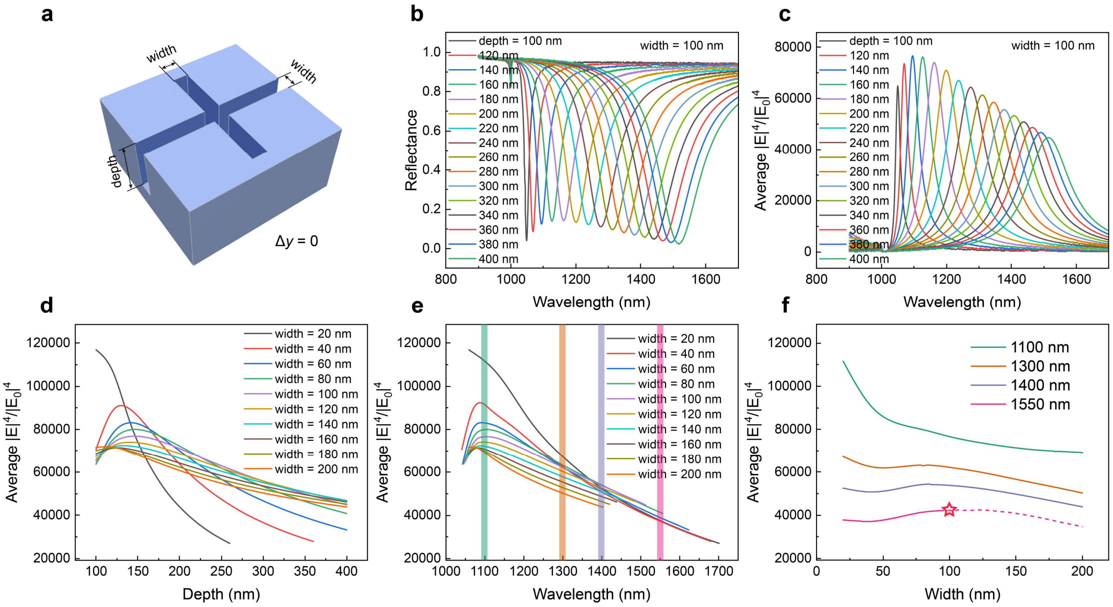


**Supplementary Fig. 3 | Performance of the structure with different paraments. a**, A schematic of the unit cell of the metasurface. $P$ = 1000 nm, $L_V$ = 800 nm. **b**,**c**, Simulated reflectivity spectra (**b**) and enhancement performance (**c**) of the structure with different depths for $w$ = 100 nm. **d**, The enhancement performance of the structure with different widths and depths. **e**, The enhancement performance of the structure with different widths and wavelengths. **f**, The summary of the structure' performance for different widths.

## Supplementary Note 4. Modulation of the asymmetry of the structure

Simulated reflectivity spectra with $\Delta y$ are shown in Fig. S4a. The short-wavelength mode is the GPP mode and the long-wavelength one is the quasi-BIC mode. As introduced in the main text, the wavelengths of the two modes gradually split up with the $\Delta y$. And the $Q$ factor of the two modes show opposite behavior with the $\Delta y$, which presents the modulating ability on the radiative loss of the structure asymmetry. Fig. S4b shows the experimental results, which is aligned with the theoretical one.

The field enhancement of the quasi-BICs can be written as $|E/E_0|^2 \propto \gamma_{rad}/[V(\gamma_{rad} + \gamma_{diss})^2]$,[2,3] where $V$ is the effective mode volume, and $\gamma_{rad}$ and $\gamma_{diss}$ represent the radiative and dissipative losses of the system. In cruciform structure, quasi-BICs can be tuned to satisfy the $\gamma_{rad} = \gamma_{diss}$ condition, thus the field enhancement can reach the maximum, as shown in Fig. S4c. Notably, in the experimental results, the optimal $\Delta y$ is larger than that of simulated one. This mainly arises from some experimentally adverse factors, e.g., during the structure fabrication process, which make actual dissipative loss $\gamma_{diss}$ be bigger than that of theory, thus more $\gamma_{rad}$ is needed to balance the $\gamma_{diss}$, leading to larger optimal $\Delta y$.

As for the length of the horizontal groove, $L_H$, it needs to be long enough to sufficiently break the symmetry of the structure, as shown in Fig. S4d. And the $L_H$ = 1000 nm is selected in our work.

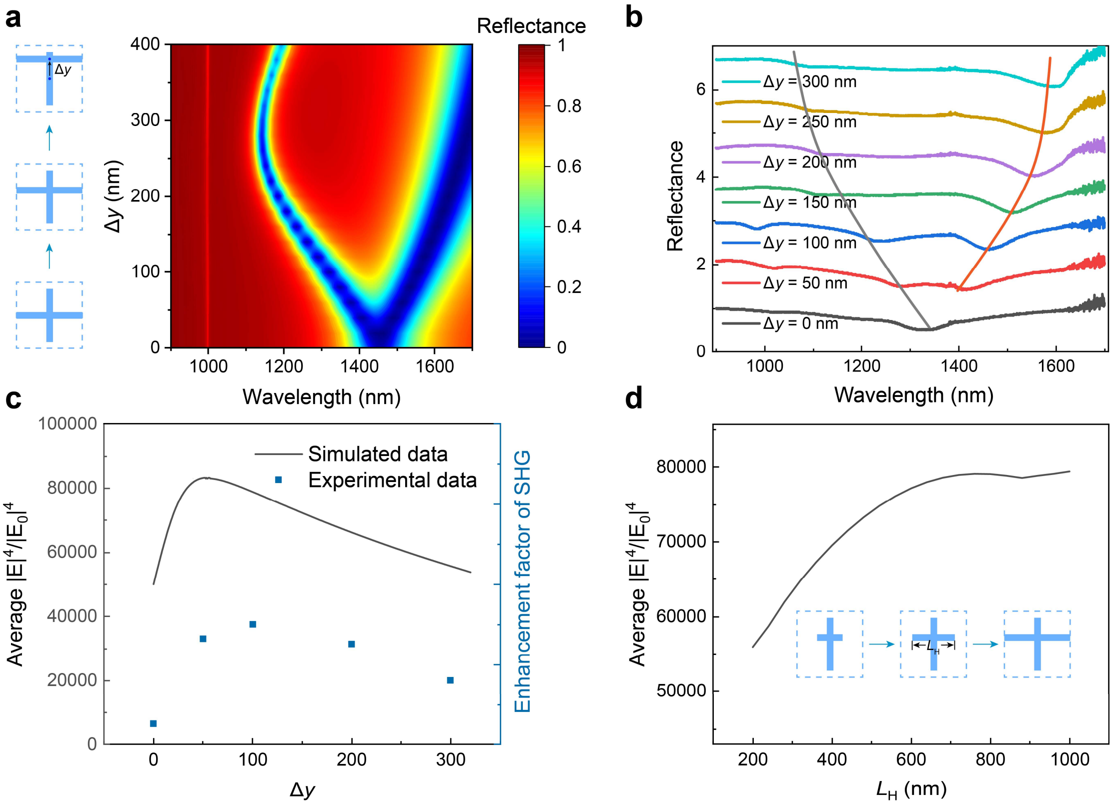


**Supplementary Fig. 4 | The influence of the horizontal groove to the quasi-BIC mode. a**, Simulated reflectivity spectra with $\Delta y$. **b**, Measured reflectivity spectra with $\Delta y$. **c**, Simulated and measured largest EFs of SHG with different $\Delta y$s. **d**, The influence of the length of the horizontal groove, $L_H$ to the performance of the metasurface.

**Supplementary Note 5. Identification of the BIC mode**

As shown in Fig. S5a, Q factor of optical mode in our structure increases with the decrease of $\Delta y$, and tends to $+\infty$ at $\Delta y = 0$, demonstrating a typical BIC feature at the high-symmetry point.[6] The far-field eigenpolarization map of the resonant mode is shown in Fig. S5b, and a singularity appears at the high-symmetry point, which is another typical feature of the perfect BIC.[7]

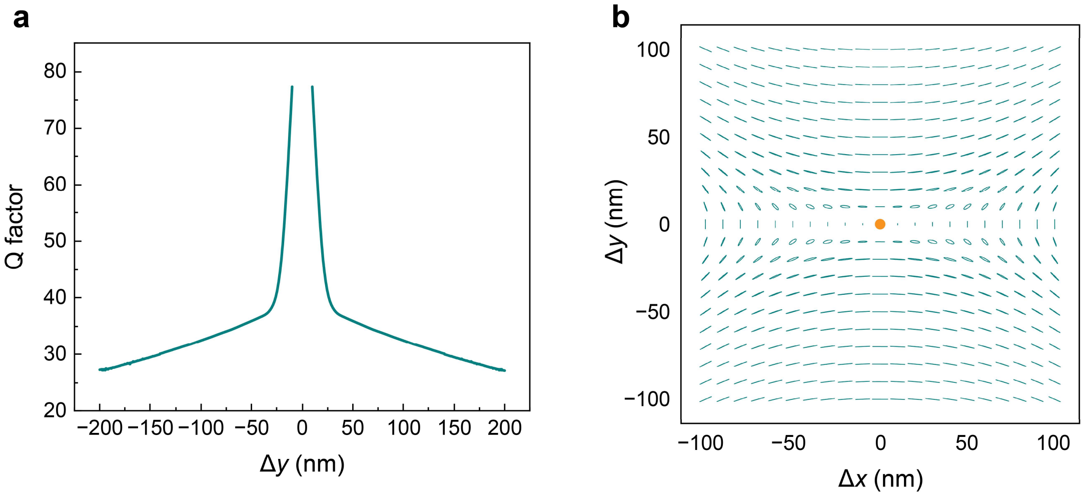


**Supplementary Fig. 5 | Evidence of perfect BIC. a**, $\Delta y$ dependent Q-factor. **b**, Asymmetry parameter dependent far-field polarization. To make $\Delta x$ meaningful, the $P$ is enlarged to 1200 nm in this simulation.

**Supplementary Note 6. Construction of anapole states**

Figure S6 shows the specific situation of the destructive interference between ED and TD, which is a direct proof of anapole states.[4,5] As shown in Fig. S6, the orange (near 1550 nm) and cyan area (near 1300 nm) represents the main resonance area of the quasi-BIC mode and the GPP mode, where the components of ED are really large. Cancelled with TD of similar magnitude and π phase difference, the component of ED+TD can be more than 2 orders of magnitude smaller than that of ED, proving the anapole effect. In practice, since the phase difference may not be exactly π and the ED+TD scattering does not vanish entirely, we define the anapole state as existing when the condition ED+TD << ED, TD is satisfied.

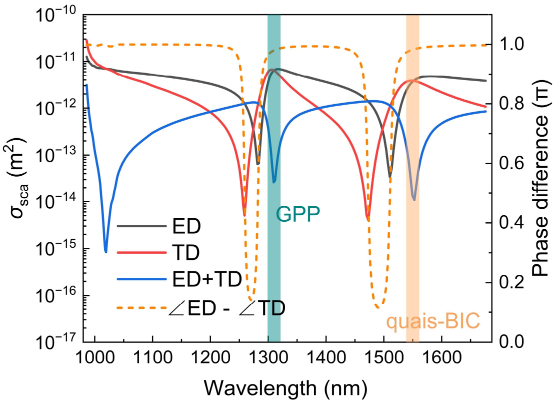


**Supplementary Fig. 6 | Scattering cross section of ED, TD and ED+TD and the phase difference between the ED and TD.** At the resonance wavelengths (cyan and orange areas), the ED and TD cancel each other the most and a minimal ED+TD is achieved.

**Supplementary Note 7. Electric properties of $MoS_2$, $WSe_2$ monolayers and the heterostructure**

In order to probing the basic electric properties of the 2D materials, the electric measurements are carried out, as shown in Fig. S7. The $MoS_2$, $WSe_2$ monolayers and the $MoS_2/WSe_2$ heterostructure are transferred to connect the electrodes on the $SiO_2/Si$ substrate, and the gate voltage is applied from the Si at the back of the substrate. The heterostructure demonstrates a better conductivity from Fig. S7a. Figure S7b shows the gating response of the three conditions. It can be found that the $MoS_2$ and $WSe_2$ monolayers are *n*-doped and *p*-doped, and the heterostructure mainly shows the hole-dominated behavior.

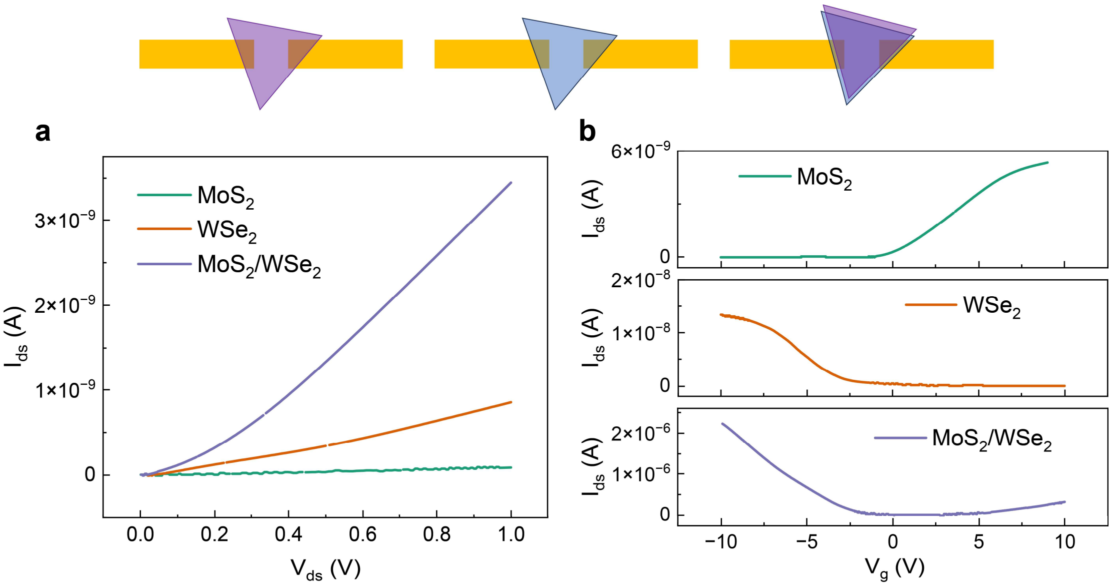


**Supplementary Fig. 7 | Electric measurements of single $MoS_2$, $WSe_2$ and the heterostructure on $SiO_2/Si$. a**, $I_{ds}$-$V_{ds}$ curves of $MoS_2$, $WSe_2$ monolayers and the $MoS_2/WSe_2$ heterostructure on $SiO_2/Si$ at $V_g = 0$. **b**, Gating response of $MoS_2$, $WSe_2$ monolayers and the $MoS_2/WSe_2$ heterostructure on $SiO_2/Si$ at $V_{ds} = 1$ V.

**Supplementary Note 8. PL spectra of the 2D TMDCs**

Figure S8a shows the PL spectra of $MoS_2$, $WSe_2$ monolayers and the $MoS_2/WSe_2$ heterostructure on the $SiO_2/Si$ substrate, in which the resonance peaks correspond to the A excitons of $MoS_2$ and $WSe_2$. Except for the red and blue shifts of the PL peaks that have been mentioned in the main text, it is clearly to see that peak intensities of $MoS_2$ and $WSe_2$ are increased and reduced when $MoS_2/WSe_2$ heterostructure formed. This can be attributed to the difference of electron affinity, leading to charge transfer between $MoS_2$ and $WSe_2$.[8]

For $MoS_2/WSe_2$ heterostructure on Ag metasurface, charge transfer process happens not only at $MoS_2$-$WSe_2$ interlayer, but also $MoS_2/WSe_2$–metal interface due to the work function difference.[9] The peak wavelengths red shift for the heterostructure on the metasurface compared to that on $SiO_2/Si$, as shown in Fig. S8b. Despite that the main mode resonances of the metasurface do not overlap with the PL process, the PL intensities are enhanced for the structural surface topography.

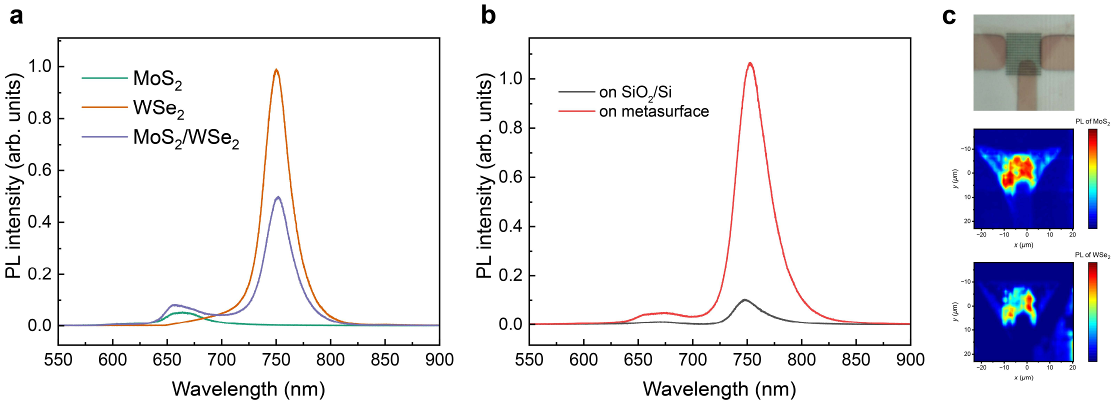


**Supplementary Fig. 8 | PL spectra of TMDCs.** **a**, PL spectra of $MoS_2$, $WSe_2$ monolayers and the $MoS_2/WSe_2$ heterostructure on the $SiO_2/Si$ substrate. **b**, The PL spectra of the $MoS_2/WSe_2$ heterostructure on the Ag metasurface compared to that on the $SiO_2/Si$ substrate. **c**, The Optical micrograph of the photodetector device, and the corresponding mapping images of the PL spectra at the exciton wavelengths of $MoS_2$ and $WSe_2$, respectively.

**Supplementary Note 9. Influence of the 2D material on the optical property of metasurface**

At the near-infrared region, coupling with TMDC monolayer, the mode wavelength will redshift ~100 nm for the changes in the dielectric environment,[10] as shown in Fig. S9. The experimental data about the metasurface shown in the main text are all measured from the metasurfaces with $MoS_2/WSe_2$ heterostructures on them.

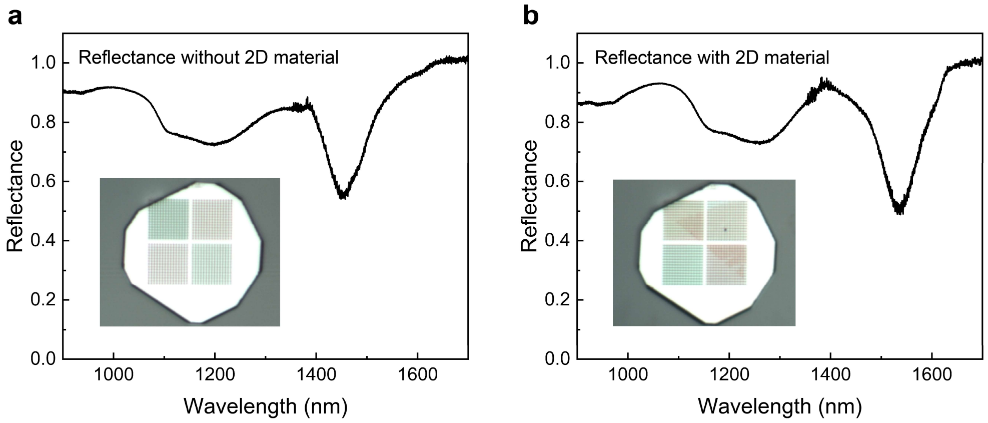


**Supplementary Fig. 9 | Reflectivity spectra of the metasurface without (a) and with (b) 2D material on it.**

**Supplementary Note 10. Advantages of the single-crystalline Ag**

The polycrystalline Ag film synthesized by magnetron sputtering is used to compare with the single-crystalline Ag here. Quantitatively, the performances of the same structure (the same as that in Fig. 2) fabricated on the single-crystalline and polycrystalline Ag are compared, as shown in Fig. S10. The SHG enhancement of the single-crystalline Ag metasurface is about 8-fold of the polycrystalline Ag metasurface (Fig. S10), proving a larger field enhancement of the single-crystalline Ag metasurface.

Therefore, single-crystalline Ag is of great significance to the device's performance.

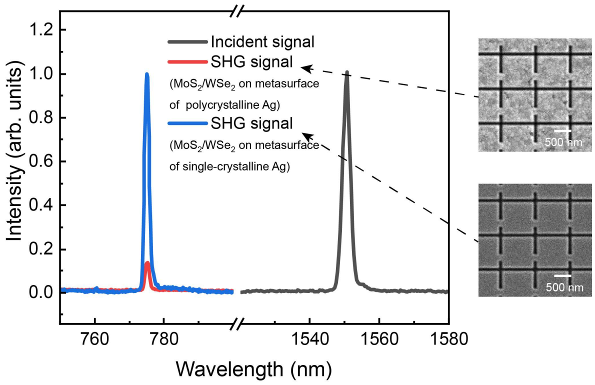


**Supplementary Fig. 10 | Comparing between polycrystalline and single-crystalline Ag metasurface.** Normalized spectra of fundamental laser (right, 1550 nm) and SHG signal (left, 775 nm) generated from the $MoS_2/WSe_2$ heterostructure on polycrystalline and single-crystalline Ag metasurface. Inset: SEM images of the structures.

**Supplementary Note 11. Consistency of the enhancement of SHG and TPA**

In our specific system, a consistent trend between the simulated field enhancement and both the SHG and TPA responses is considered. We attribute this correlation to the dominant role played by the deeply subwavelength plasmonic cavity. Regarding the phase-matching requirement for SHG, its stringency is greatly relaxed in our atomic-thickness materials compared to bulk materials or extended waveguides. The conversion efficiency here is predominantly dictated by the local field confinement and enhancement at the fundamental frequency, rather than by phase matching over a propagation length.

Therefore, while the nonlinear susceptibilities are indeed distinct, both processes share a common driving force: the local intensity at the pump frequency, which scales with $|E_\omega|^4$ in our model. Generally, the SHG power can be written as[10,11]

$$P_{\mathrm{SHG}} = \left(\chi_{eff}^{(2)}\right)^2 \times \frac{(\omega L)^2}{A\varepsilon_0 c^3} \times P_\omega^2, \qquad \text{(S1)}$$

where $P_{\mathrm{SHG}}$ represents the power of SHG, $\omega$ the angular frequency of pump laser, $L$ the length of the nonlinear material, $A$ the laser area, $\varepsilon_0$ the vacuum permittivity, $c$ the speed of light in vacuum, $P_\omega$ the power of the pump laser, and $\chi_{eff}^{(2)}$ the effective second-order nonlinear coefficient at the angular frequency of $\omega$. The above equation is derived based on some assumptions, especially, the phase-matching condition. Comparing the same materials on flat Ag and on Ag metasurface under the same optical system, $\omega$, $L$, $A$, and $\chi_{eff}^{(2)}$ are all unchanged, and the only variable is $P_\omega$. With $P_\omega \propto |E_\omega|^2$, the enhancement of $P_{\mathrm{SHG}}$ can be written as $\mathrm{EF_{SHG}} = P_{en}^2/P_0^2 = |E_{en}/E_0|^4$, where the subscript $_{\mathrm{en}}$ represents the enhanced parameter, and subscript $_0$ that before enhancement. Note that, without resonance at the emitting wavelength, the emitting enhancements of materials on flat Ag and on Ag metasurface are seen as the same.

TPA carrier production rate can be written as

$$G_{\mathrm{TPA}} = \frac{\beta}{2\hbar\omega A^2} \times P_\omega^2, \qquad \text{(S2)}$$

where $G_{\mathrm{TPA}}$ represents the TPA carrier production rate, $\beta$ the TPA coefficient, $\hbar\omega$ the energy of a photon. Similar to Eq. (S1), the enhancement of $G_{\mathrm{TPA}}$ can be written as $\mathrm{EF_{TPA}} = P_{en}^2/P_0^2 = |E_{en}/E_0|^4$.

Then, it can be obtained that $\mathrm{EF_{SHG}} = \mathrm{EF_{TPA}} = |E_{en}/E_0|^4$.

**Supplementary Note 12. SHG spectra for heterostructure on metasurface**

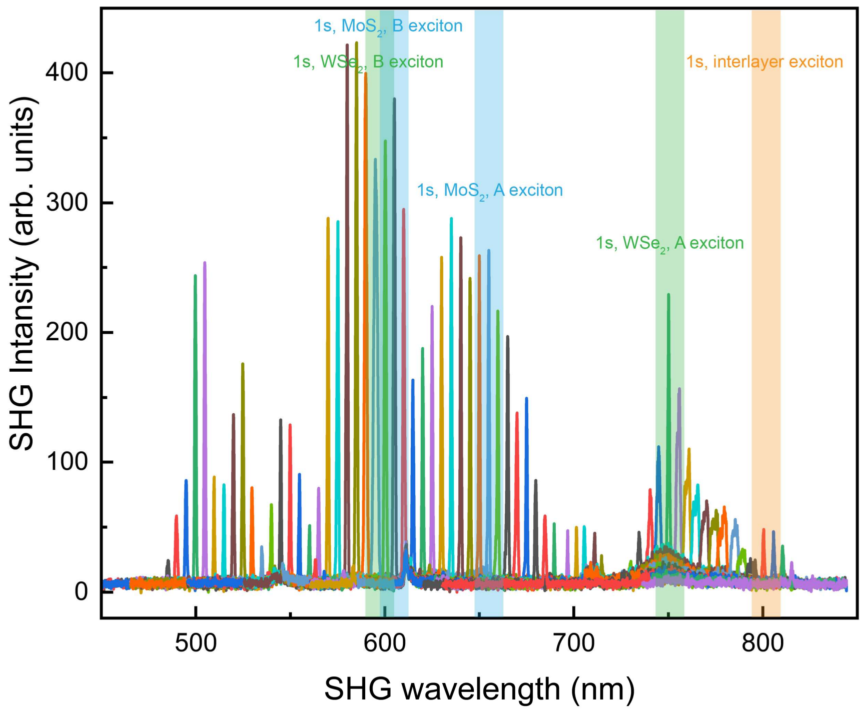


**Supplementary Fig. 11 | SHG spectra of the $MoS_2$/$WSe_2$ heterostructure.** The marked peaks are the resonances of 1s states in the heterostructure, and others reflects the dark states in the heterostructure.[13]

**Supplementary Note 13. Power-dependent optical and photoelectric signals**

Figure S12a shows the power-dependent SHG signals from heterostructure on the Ag metasurface. The slope ~2.02 of the fitting line, i.e., quadratic dependence on input power, is in agreement with the second-order nature of the nonlinear process.[11]

For the photocurrent, the slope of the measured data is between 1 and 2, as shown in Fig. S12b. The data can be fitted with the equation:

$$I_{\text{phc}} = k_1 \times P^{\alpha 1} + k_2 \times P^{\alpha 2}, \qquad \text{(S3)}$$

where the first term on the right side represents the photocurrent from directly generated hot holes via Ag metasurface, and the second term represents that from TPA photocarrier generation. The power exponents are calculated as $\alpha_1 = 0.86$ and $\alpha_2 = 1.94$, corresponding to linear and nonlinear processes respectively, as shown in Fig. S12b. Note that $\alpha_1 < 1$ and $\alpha_2 < 2$, which can be attributed to the filling of trap-states with the increase of the input power.[14,15]

For a small input power ($P < 100$ μW), $k_1 \times P^{\alpha 1} >> k_2 \times P^{\alpha 2}$. Then, $I_{\text{phc}} \approx k_1 \times P^{0.86}$, suggesting that the metasurface-induced hot-hole mechanism dominate the device performance. For a large input power ($P > 100$ μW), the nonlinear process is predominant, i.e., $I_{\text{phc}} \approx k_2 \times P^{1.94}$, proving that the most photocurrent comes from the TPA photocarrier generation. Under the laser power of 2 mW, the contribution of the TPA photocarrier can be calculated by $(k_2 \times P^{\alpha 2})/I_{\text{phc}} \approx 90\%$.

Besides, the measured data begins to deviate from the fitting curve at high input powers ($P > 2$ mW), indicating the onset of device saturation.

Under continuous-wave (CW) illumination, the power-dependent photocurrent is also carried out, as shown in the inset of Fig. S12b. At low incident power (< 100 μW), the photoresponse under both CW and pulsed illumination are comparable for the domination of the linear mechanism. As the incident power increases (> 100 μW), the response under pulsed illumination surpasses that under CW illumination. This is because the high peak intensity of the pulsed laser strongly promotes the nonlinear TPA process, whereas under CW illumination the response remains primarily linear due to the lower instantaneous power.

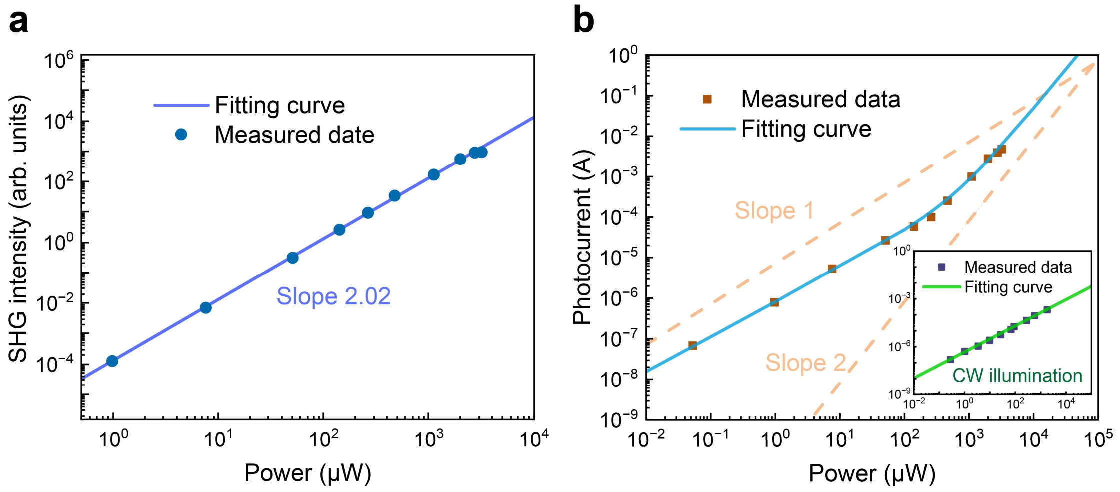


**Supplementary Fig. 12 | SHG intensity and photocurrent of the device as a function of laser power at 1550 nm. a**, SHG intensity of $MoS_2/WSe_2$ heterostructure on the metasurface with input power on a log-log scale under the excitation wavelength

of 1550 nm. **b**, Photocurrent of $MoS_2/WSe_2$ heterostructure on the metasurface by varying incident power at $V_{ds} = 1$ V, $V_g = -5$ V under the excitation wavelength of 1550 nm. The photocurrent can be fitted as $I_{phc} = k_1 \times P^{\alpha 1} + k_2 \times P^{\alpha 2}$, where $\alpha_1 = 0.86$, $\alpha_2 = 1.94$, $k_1 \approx 8.12 \times 10^{-7}$ A·μW$^{-\alpha 1}$, $k_2 \approx 7.97\times 10^{-10}$ A·μW$^{-\alpha 2}$. Inset: Photocurrent of the device as a function of laser power at 1550 nm under CW illumination.

**Supplementary Note 14. Component analysis of the enhanced photocurrent**

As mentioned above, the enhanced photocurrent can be divided into the exciton-related component and the direct absorbed component. In addition to power-dependent measurements, the former can be calculated by the SHG EF of the device for that the TPA enhancement can be considered the same with SHG enhancement. The signals from $MoS_2/WSe_2$ heterostructure on the metasurface and on the single-crystalline Ag are compared to exclude the difference between dielectric and metal substrate. Then, the enhanced photocurrent from the TPA enhancement can be obtained as $I_{Ex}$, and the component from the direct absorption of Ag metasurface can be obtained as $I_{Ag} = I_{phc} - I_{Ex}$, as shown in Fig. S13.

The result is based on the assumption that the enhancement of TPA is equal to that of exciton-related photocurrent. Actually, the actual $I_{Ex}$ should be larger than that shown in Fig. S13. The structural surface topography also help the energy of excitons easier to decaying to the channels like SPPs and electron-hole excitation, which furtherly boost the recycled hot holes mentioned in the main text. But the impact of this part is difficult to estimate.

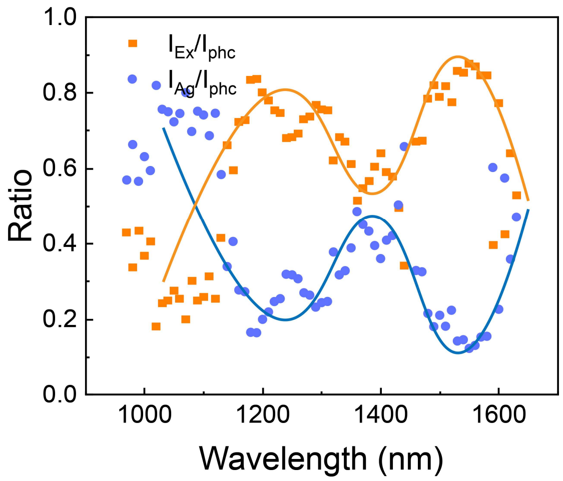


**Supplementary Fig. 13 | The ratio of exciton-related component and the direct absorbed component of the photocurrent for $MoS_2/WSe_2$ heterostructure on the metasurface.**

## Supplementary Note 15. Noise measurement and detectivity assessment

In order to calculate the NEP of the photodetector, we measure the dark current of the device with a source-drain voltage $V_{ds} = 1$ V and back-gate voltage $V_g = -5$ V. After fast Fourier transform, the noise power spectrum can be obtained, as shown in Fig. S14a. At 1 Hz, we get the noise current spectral density of the detector $S(f = 1\text{Hz}) = 1.21 \times 10^{-12}$ A/Hz$^{1/2}$. With the responsivity R = 1.35 A/W we extract a NEP of $8.96 \times 10^{-13}$ W/Hz$^{1/2}$. The specific detectivity $D^*$ in this circumstance can be calculated as follows[16–18]

$$D^* = \frac{\sqrt{AB}}{\text{NEP}} = R\frac{\sqrt{A}}{S}, \qquad \text{(S4)}$$

where $R$ is the responsivity, $A$ the active area of the detector, $B$ the noise bandwidth, and $S$ the noise current spectral density of the detector. With the measured $R$ in the NIR-II window as shown in Fig. 2e, the $D^*$ at different wavelengths can be obtained, as shown in Fig. S14b. At 1550 nm, the $D^*$ reaches $2.08 \times 10^8$ Jones. The EQE of the device can be estimated by the formula EQE = $hcR/e\lambda$, where $h$, $c$, $R$, $e$, and $\lambda$ represent the Planck constant, the speed of light in the vacuum, responsivity, elementary charge and wavelength, respectively. Figure S14c shows the EQE of the photodetector in the NIR-II window, reaching 108% at 1550 nm. The EQE that exceeds 100% is attributed to the large photoconductive gain of the device.

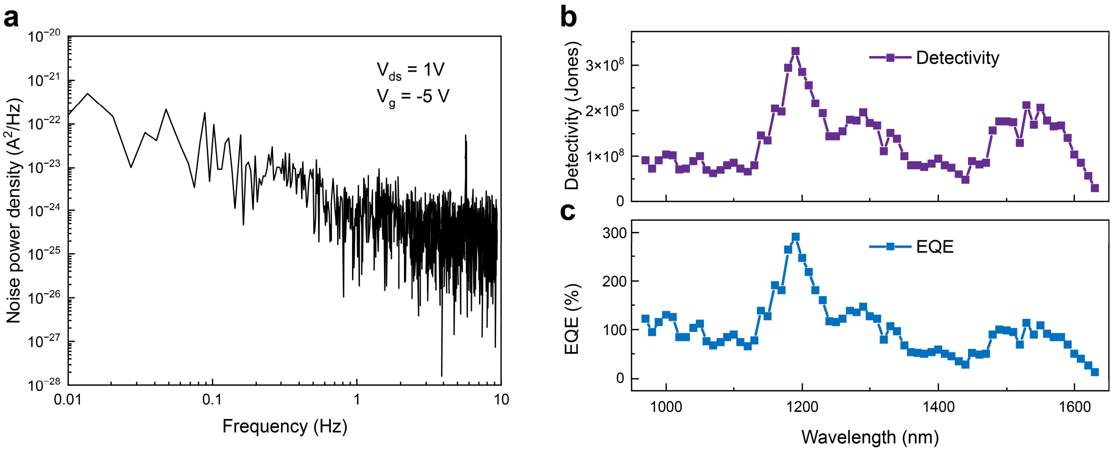


**Supplementary Fig. 14 | a,** Noise power density of the dark current of the photodetector measured for $V_{ds} = 1$ V and $V_g = -5$ V, corresponding to the same conditions in which we measured its responsivity. **b,** Specific detectivity $D^*$ and **c**, EQE of the photodetector.

## Supplementary Note 16. Photoconductive gain mechanism

Figure S15 shows the $V_{ds}$-resolved and time-resolved photoresponse of photodetector under input laser of 1550 nm. The photocurrent shows a linear dependence on the bias voltage (Fig. S15a), suggesting higher responsivity can be readily achieved by applying a larger bias voltage, which indicates a larger photoconductive gain. The gain of the detector is defined as $G = \tau_{life}/\tau_{transit}$,[17] where $\tau_{life}$ is the lifetime of the carrier and $\tau_{transit}$ is the transiting time of the carrier. we first consider the $\tau_{transit}$, which is inversely proportional to the electron mobility and can be defined as $\tau_{transit} = L^2/\mu V_{ds}$, where $L$ is length of the channel, $\mu$ is the mobility and $V_{ds}$ is the applied bias, which is calculated as $\tau_{transit} \sim 11$ ns. From the temporal response of the photodetectors (shown in Fig. S15b), the $\tau_{life}$ is estimated on the order of 300 ms. As a result, multiple electrons are recirculated in the heterostructure channel following a single electron-hole photo-generation, leading to a photoconductive gain on the order of $10^7$.

In such a regime where $\tau_{life} >> \tau_{transit}$, the responsivity speed is primarily limited by $\tau_{life}$. A shorter $\tau_{life}$ leads to faster response, whereas a longer $\tau_{life}$ enhances the photoconductive gain. This highlights a fundamental trade-off between the response speed and the gain in the photoconductive operation of the detector.

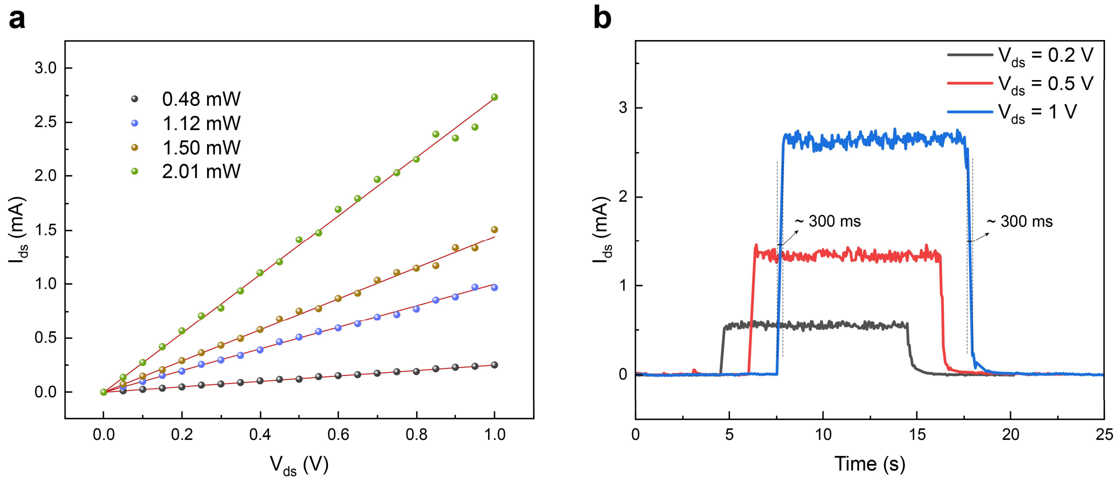


**Supplementary Fig. 15 | a,** The magnitude of the photocurrent increases linearly with source-drain bias voltage under input laser (1550 nm) of different power. $V_g$ = -5 V. **b,** Time-resolved photoresponse of the device, recorded for different values of bias voltage $V_{ds}$ with input power (1550 nm) of 2 mW. $\tau_{rise} \approx \tau_{decay} \approx 300$ ms.

**Supplementary Note 17. Regulation of Schottky barrier by gate voltage**

To investigate the gate-tunable Schottky barrier at the Ag/$WSe_2$ interface, we decomposed the photocurrent by leveraging the distinct power dependencies of the two dominant generation mechanisms: hot-carrier injection (linear) and TPA (quadratic). As detailed in Supplementary Note 13, the photocurrent of our device can be written as Eq. (S3): $I_{phc} = k_1 \times P^{\alpha 1} + k_2 \times P^{\alpha 2}$, where the exponent $\alpha_1 \sim 1$ corresponds to the linear hot-carrier process, and $\alpha_2 \sim 2$ corresponds to the nonlinear TPA process.

We measured the power-dependent photocurrent under different $V_g$, as shown in Fig. S16a. By fitting the data to Eq. (S3), we extracted the coefficients $k_1$ and $k_2$, which quantitatively represent the efficiency of the hot-carrier injection and TPA contributions, respectively (Fig. S16b). The $V_g$ dependence of $k_2$ mirrors the trend of the dark $I_{ds}$ - $V_g$ curve in Fig. 1g, which is governed by the electrostatic doping of the heterostructure bulk. In contrast, the strong modulation of $k_1$ with $V_g$ directly evidences the gate control over the Schottky barrier height $\Phi_B$. At $V_g = 0$ V, the small $k_1$ indicates a high $\Phi_B$, which strongly suppresses hot-hole injection. When a negative gate voltage ($V_g$ = -5 V) is applied, $k_1$ increases substantially, signifying a lowering of $\Phi_B$ that efficiently promotes the injection of hot holes from the Ag metasurface into $WSe_2$. This delineation not only confirms the gate-tunable nature of the interface barrier but also explicitly demonstrates how its modulation dictates the hot-carrier contribution to the overall photoresponse, solidifying our understanding of the device's operational principle.

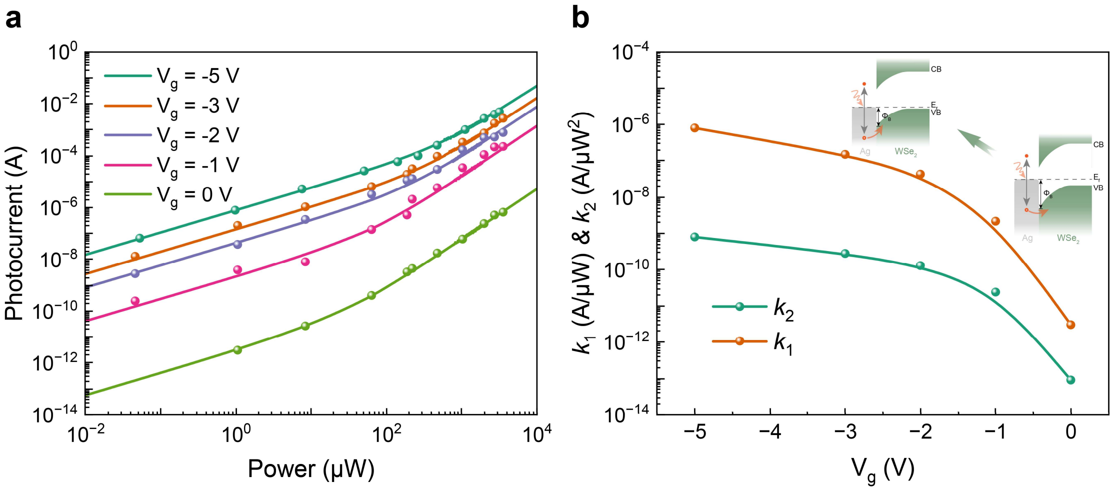


**Supplementary Fig. 16 | a**, Photocurrent of the device as a function of laser power at 1550 nm under different $V_g$. The photocurrent can be fitted as $I_{phc} = k_1 \times P^{\alpha 1} + k_2 \times P^{\alpha 2}$, where $\alpha_1 \sim 1$, $\alpha_2 \sim 2$. **b**, Fitted $k_1$ and $k_2$ under different $V_g$. The increased $k_1$ with a larger negative $V_g$ indicates the decreased Schottky barrier under negative $V_g$.

## Supplementary Note 18. Polar plots of SHG

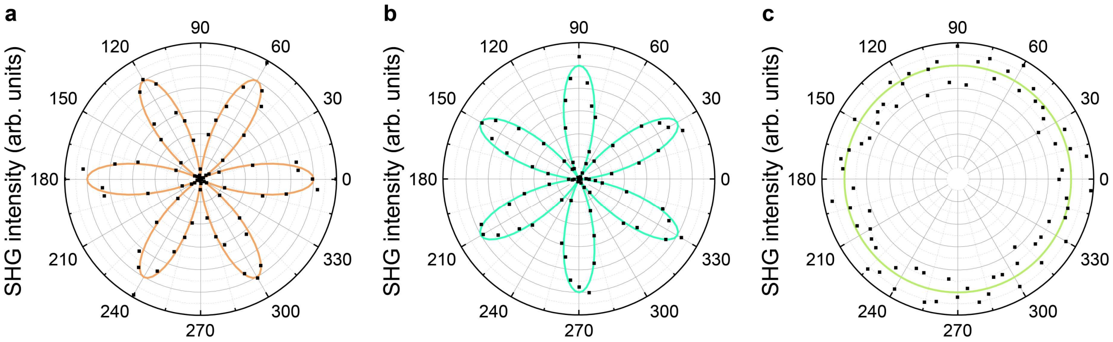


**Supplementary Fig. 17 | Polar plots of the SHG of TMDCs a,b**, Polar plots of the normalized perpendicular (**a**) and parallel (**b**) component of SHG intensity from the $MoS_2/WSe_2$ heterostructure on the $SiO_2/Si$ substrate with the entire 360° rotation of the sample. **c**, The normalized summation of the polar plots in (**a**) and (**b**). The angle is defined by the angle between the polarization and the edge of TMDCs

## Supplementary Note 19. Regulation of the vertically polarized mode

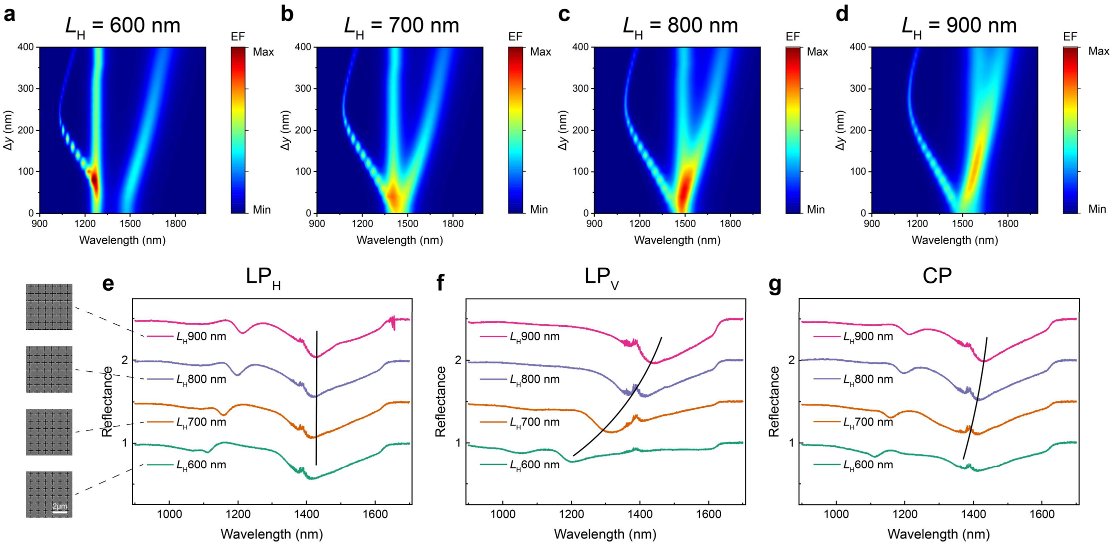


**Supplementary Fig. 18 | Regulation of the mode wavelengths within the metasurface with different $L_H$s. a–d**, Simulated EF spectra of the metasurface under the circular polarized light of different $L_H$ = 600 (**a**), 700 (**b**), 800 (**c**), and 900 (**d**) nm. The vertically polarized mode redshifts with the $L_H$. And the two horizontally polarized modes are nearly not influenced by the $L_H$. **e–g**, Measured reflectivity spectra of the metasurface of different $L_H$s under the input light of horizontal linear (**e**), vertical linear (**f**) and circular (**g**) polarization. The SEM images are listed by the side.

**Supplementary Note 20. Chiral measurement of the structure with mirror symmetry**

For structure with mirror symmetry, it shows the same mode resonance and photocurrent under LCP and RCP illumination despite the different field intensity distributions, as shown in Fig. S19.

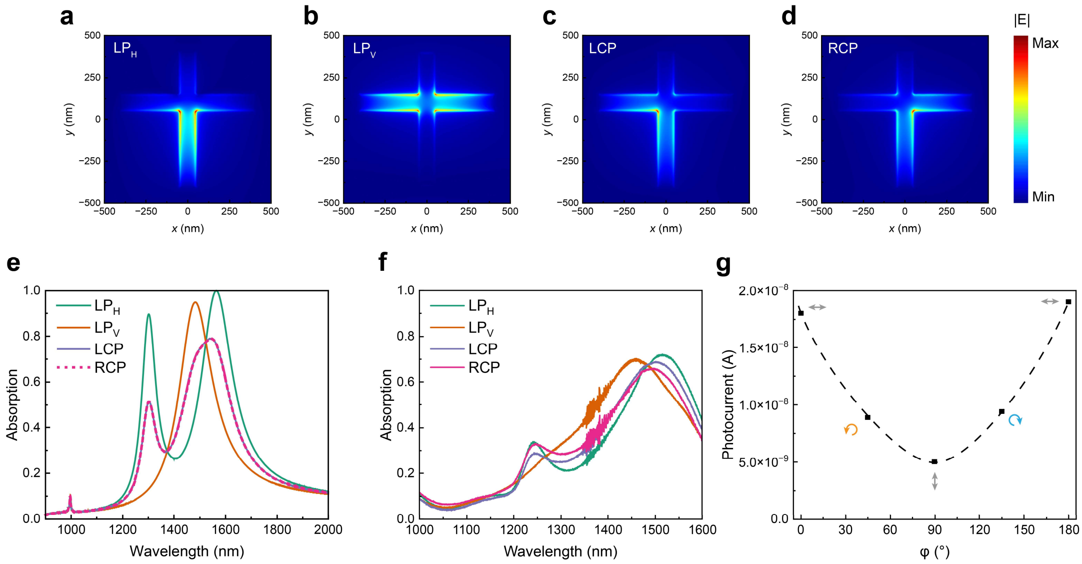


**Supplementary Fig. 19 | Properties of the structure with $\Delta x = 0$, $\Delta y = 100$ nm. a–d,** Simulated electric field distributions of the metasurface under the input light of horizontal linear (**a**), vertical linear (**b**) and left circular (**c**) and right circular (**d**) polarization. **e**,**f**, Simulated (**e**) and measured (**f**) reflectivity spectra of the structure under the input light of different polarizations. **g**, Photocurrents of the heterostructure on the metasurface under the input light of different polarizations at $V_{ds} = 1$ V, $V_g = 0$ V.

## Supplementary Note 21. Influence of the angle of the cruciform structures

As another way to break the mirror symmetry, the cruciform structures can be rotated, as shown in Fig. S20. However, the rotating of the structures only changes the wavelength of mode and does not show strong chirality. Therefore, such plan was not adopted by us.

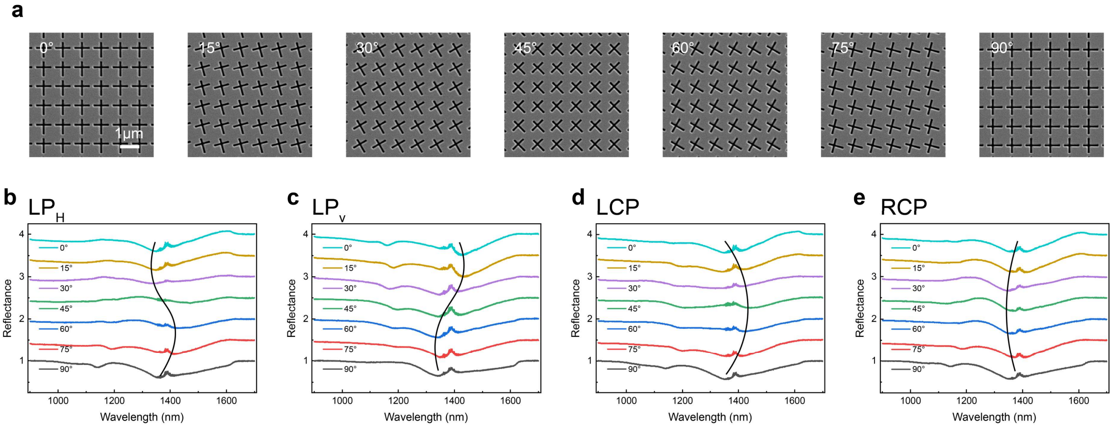


**Supplementary Fig. 20 | Measurements of the structure with different angles, $L_H$ = 800 nm. a**, SEM images of the structures with different angles. **b–e**, Measured reflectivity spectra of the metasurface with cruciform structures of different angles under the input light of horizontal linear (**b**), vertical linear (**c**) and left circular (**d**) and right circular (**e**) polarization.

## Supplementary Note 22. Modulation of the mirror asymmetry

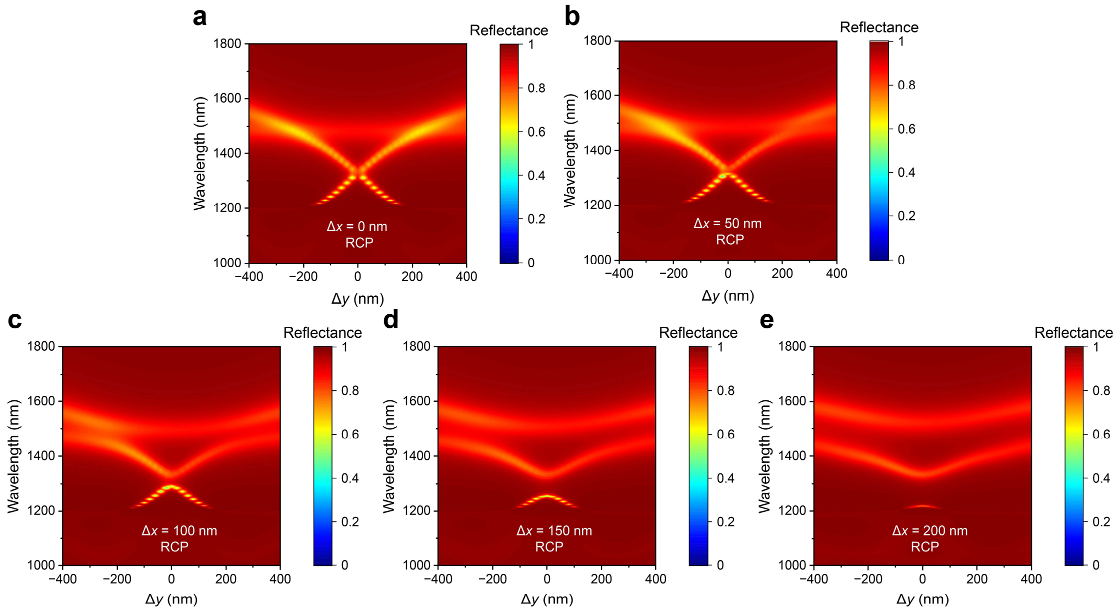


**Supplementary Fig. 21 | Reflectivity spectra of the structure with varying Δ*x*s**. **a**–**e**, Calculated reflection spectra under RCP illumination with structural asymmetry Δ*y*, for Δ*y* = 0 (**a**), 50 (**b**), 100 (**c**), 150 (**d**), 200 (**e**) nm. And the spectra under LCP illumination are mirror symmetrical with **a**–**e**.

## Supplementary Note 23. Angle-resolved spectra of the chiral structure

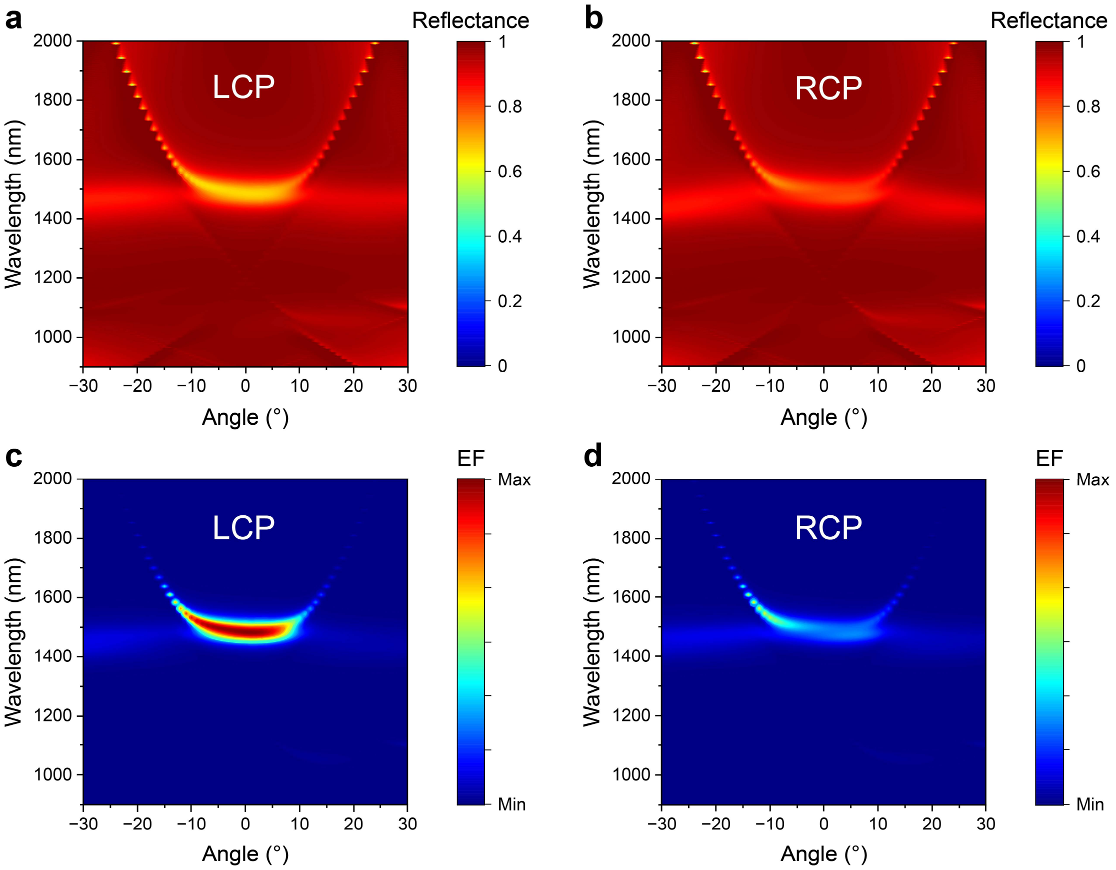


**Supplementary Fig. 22 | Angle-resolved reflectivity and EF spectra of the structure in Fig. 4, Δ*x* = 50 nm and Δ*y* = 250 nm**. **a**,**b**, Calculated angle-resolved white light reflection spectra for the LCP (**a**) and RCP (**b**) illumination. **c**,**d**, Calculated angle-resolved SHG/TPA EF spectra for the LCP (**c**) and RCP (**d**) illumination.

**Supplementary Note 24. Multipole decomposition of the chiral-resolved structure**

The evidence, presented in Fig. 4g,h, shows a strong resonance and field enhancement only under LCP. The multipole decomposition in Fig. S23a under LCP illumination reveals the key signature of the anapole state: a destructive interference between ED and TD, alongside the hierarchy of EQ > EO > ED+TD. In contrast, the RCP response (Fig. S23b) does not fulfill this condition. The anapole state is absent, and the mode is primarily dominated by the ED, leading to a weak photoresponse.

Therefore, the symmetry breaking does not destroy the anapole state but rather makes it chiral-dependent, which is the very origin of the chiral-resolved photoresponse we report.

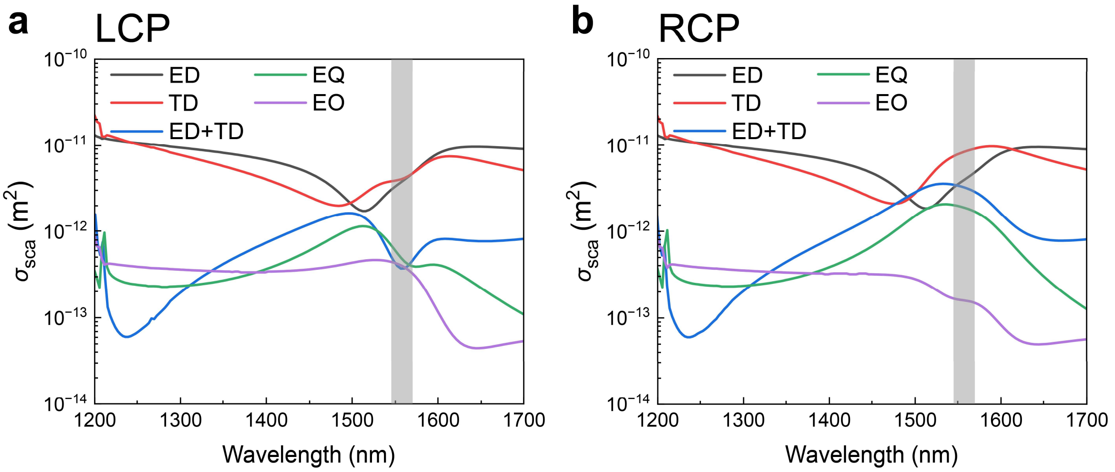


**Supplementary Fig. 23 | Multipole decomposition of the optical mode of chiral-resolved metasurface in Fig. 4 under LCP (a) and RCP (b) illumination.**

## Supplementary Note 25. Chiral measurements of SHG

Figure S24a shows the wavelength-dependent DCP of SHG, which is consistent to the discrimination ratio shown in Fig. 4h. For the ellipticity angle dependent SHG polar diagram, the data is measured by rotating the quarter-wave plate after the horizontal and vertical linear polarized light, respectively, as shown in Fig. S24b–c. And the diagram in Fig. 4i is achieved by the same method.

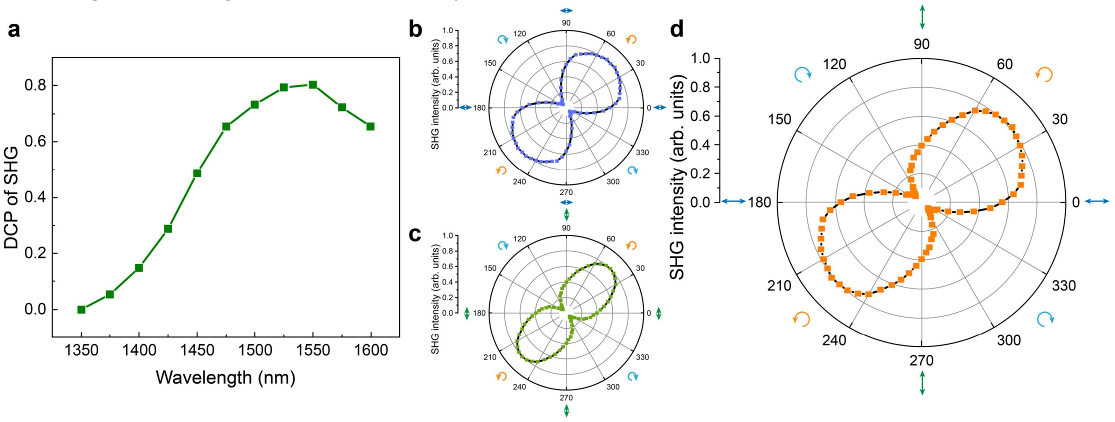


**Supplementary Fig. 24 | Chiral measurements of SHG for the heterostructure on the structure in Fig. 4, Δ*x* = 50 nm and Δ*y* = 250 nm**. **a**, The DCP of SHG for varying wavelengths. **b**,**c**, Ellipticity angle dependent responsivity polar diagrams at the wavelength of 1550 nm measured by rotating the quarter-wave plate after the horizontal (**b**) and vertical (**c**) linear polarized light. **d**, The data combination of that in (**b**) and (**c**).

**Supplementary Note 26. Comparison of photoresponse performances**

A comprehensive performance comparison (Table S1) confirms that our $MoS_2/WSe_2$-based photodetector with an Ag metasurface achieves a high responsivity of 1.35 A/W at 1550 nm, outperforming most counterparts in similar spectral ranges while also exhibiting broadband response. Critically, the plasmonic metasurface, engineered to support anapole states, enables a substantial photocurrent enhancement of $\sim 5 \times 10^4$, highlighting the distinct advantage of our design. Furthermore, the metasurface can be tailored to support chiral-resolved photodetection, extending both the functionality and applicability of the proposed structure.

**Supplementary Table 1 | Photoelectric performances of structure-enhanced near-infrared photodetectors.**

| Material | Structural type | Wavelength (nm) | Delay (ms) | Responsivity (A/W) | EF | Reference |
|---|---|---|---|---|---|---|
| $MoS_2/WSe_2$ | Ag metasurface | 1550 | 300 | 1.35 | 50000 | This work |
| $MoS_2$ | Au nanoparticles | 980 | 2.6 | $6.4 \times 10^{-2}$ | 14 | 19 |
| $MoS_2$ | Ag nanoparticles | 1550 | – | $5.39 \times 10^{-4}$ | 2.07* | 20 |
| $MoS_2$ | Au resonant wires | 1070 | 216000 | 5.2 | – | 21 |
| Si/ Gr | waveguide | 1550 | – | 0.37 | 9.25* | 22 |
| BP | Plasmonic bowtie antenna | 1550 | – | $1.42 \times 10^{-2}$ | 4 | 23 |
| Colloidal QDs | Au metasurface | 1530 | $2.32 \times 10^{-3}$ | $8.2 \times 10^{3}$ | 10 | 24 |
| Si/ Gr | Au metasurface | 1550 | $8 \times 10^{-3}$ | $1.5 \times 10^{-5}$ | – | 25 |

*Calculated from the data in the corresponding reference. Gr: Graphene; BP: black phosphorus; QD: Quantum Dot.

**Supplementary Note 27. Photoelectric measurement platform**

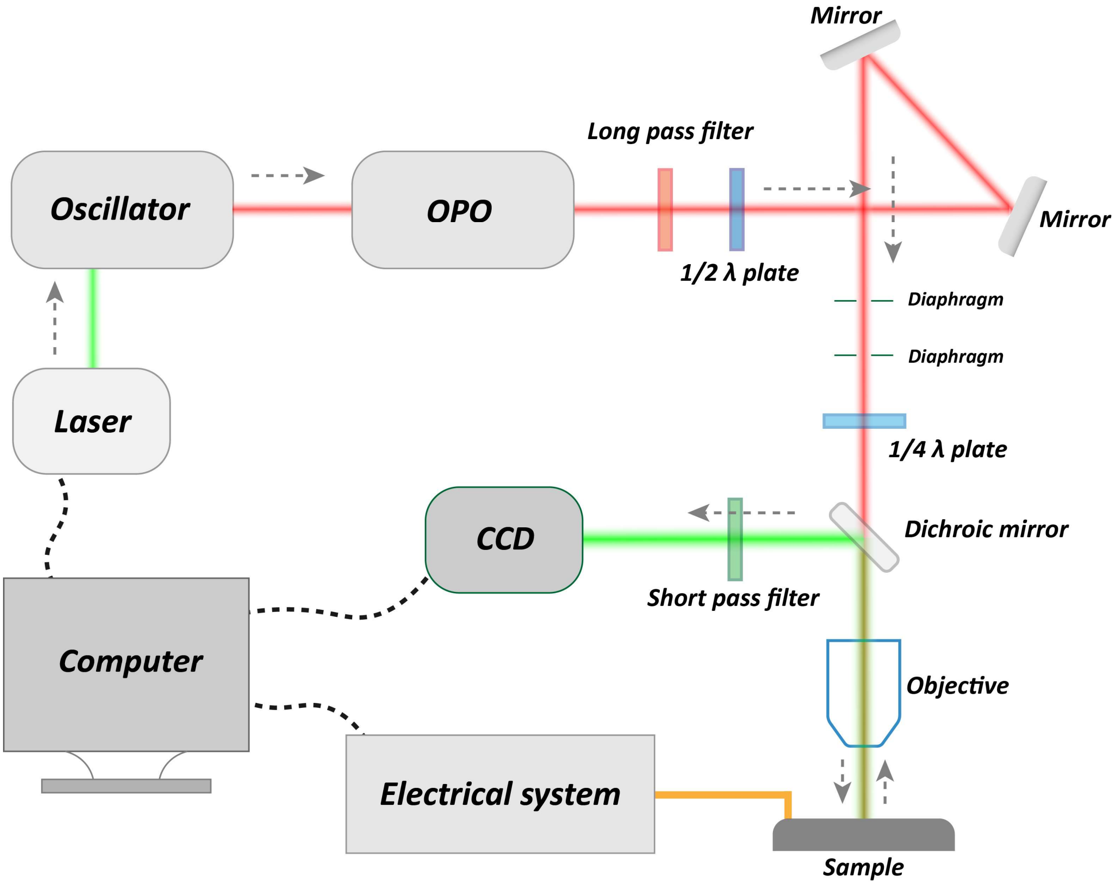


**Supplementary Fig. 25 | A layout of home-built platform for photoelectric measurements.**

**Supplementary References**


1. Qin, K. et al. Strongly coupled Raman scattering enhancement revealed by scattering-type scanning near-field optical microscopy. *Nanophotonics* **12**, 1857−1864 (2023).
2. Liang, Y. et al. Bound states in the continuum in anisotropic plasmonic metasurfaces. *Nano Lett.* **20**, 6351−6356 (2020).
3. Maier, S. A. Plasmonic field enhancement and SERS in the effective mode volume picture. *Opt. Express* **14**, 1957–1964 (2006).
4. Koshelev, K., Favraud, G., Bogdanov, A., Kivshar, Y. & Fratalocchi, A. Nonradiating photonics with resonant dielectric nanostructures. *Nanophotonics* **8**, 725−745 (2019).
5. Baryshnikova, K. V., Smirnova, D. A., Luk'yanchuk, B. S. & Kivshar, Y. S. Optical anapoles: Concepts and applications. *Adv. Optical Mater.* **7**, 1801350 (2019).
6. Koshelev, K., Lepeshov, S., Liu, M., Bogdanov, A. & Kivshar, Y. Asymmetric metasurfaces with high-Q resonances governed by bound states in the continuum. *Phys. Rev. Lett.* **121**, 193903 (2018).
7. Hsu, C. W., Zhen, B., Stone, A. D., Joannopoulos, J. D. & Soljačić, M. Bound states in the continuum. *Nat. Rev. Mater.* **1**, 16048 (2016).
8. Kim, J. Y. et al. Energy and charge transfer effects in two-dimensional van der Waals hybrid nanostructures on periodic gold nanopost array. *Appl. Phys. Lett.* **112**, 193101 (2018).
9. Zhang, L. et al. Modulated interlayer charge transfer dynamics in a monolayer TMD/metal junction. *Nanoscale* **11**, 418−425 (2019).
10. Zhang, Q.-H. et al. Boosting optical nonlinearity of van der Waals materials with high-order multipoles. *Laser & Photonics Rev.* **19**, 2401850 (2025).
11. Shen, Y. R. The Principles of Nonlinear Optics. Wiley-Interscience: New York, 1984.
12. Xu, C. & Webb, W. W. Measurement of two-photon excitation cross sections of molecular fluorophores with data from 690 to 1050 nm. *J. Opt. Soc. Am. B* **13**, 481−491 (1996).
13. Wang, G. et al. Giant enhancement of the optical second-harmonic emission of $WSe_2$ monolayers by laser excitation at exciton resonances. *Phys. Rev. Lett.* **114**, 097403 (2015).
14. Buscema, M. et al. Photocurrent generation with two-dimensional van der Waals semiconductors. *Chem. Soc. Rev.* **44**, 3691 (2015).
15. Xiong, Y.-F., Chen, J.-H., Lu, Y.-Q., & Xu, F. Broadband optical-fiber-compatible photodetector based on a graphene-$MoS_2$-$WS_2$ heterostructure with a synergetic photogenerating mechanism. *Adv. Electron. Mater.* **5**, 1800562 (2019).
16. Liu, C.-H., Chang, Y.-C., Norris, T. B. & Zhong, Z. Graphene photodetectors with ultra-broadband and high responsivity at room temperature. *Nat. Nanotech.* **9**, 273−278 (2014).
17. Lopez-Sanchez, O., Lembke, D., Kayci, M., Radenovic, A., & Kis, A. Ultrasensitive photodetectors based on monolayer $MoS_2$. *Nat. Nanotech.* **8**,

497−501 (2013).
18. Huo, N. & Konstantatos, G. Ultrasensitive all-2D $MoS_2$ phototransistors enabled by an out-of-plane $MoS_2$ PN homojunction. *Nat. Commun.* **8**, 572 (2017).
19. Guo, J. et al. Near-infrared photodetector based on few-layer $MoS_2$ with sensitivity enhanced by localized surface plasmon resonance. *Appl. Surf. Sci.* **483**, 1037–1043 (2019).
20. Park, M. J., Park, K. & Ko, H. Near-infrared photodetector achieved by chemically-exfoliated multilayered $MoS_2$ flakes. *Appl. Surf. Sci.* **448**, 64–70 (2018).
21. Wang, W. et al. Hot electron-based near-infrared photodetection using bilayer $MoS_2$. *Nano Lett.* **15**, 7440–7444 (2015).
22. Goykhman, I. et al. On-chip integrated, silicon–graphene plasmonic Schottky photodetector with high responsivity and avalanche photogain. *Nano Lett.* **16**, 3005–3013 (2016).
23. Venuthurumilli, P. K., Ye, P. D. & Xu, X. Plasmonic resonance enhanced polarization sensitive photodetection by black phosphorus in near infrared. *ACS Nano* 2018, **12**, 4861.
24. Đorđević, N. et al. Metasurface colloidal quantum dot photodetectors. *ACS Photonics* **9**, 482–492 (2022).
25. Li, L. et al. Monolithic full-stokes near-infrared polarimetry with chiral plasmonic metasurface integrated graphene–silicon photodetector. *ACS Nano* **14**, 16634–16642 (2020).
